# Preschoolers prefer to learn causal information

**DOI:** 10.3389/fpsyg.2015.00060

**Published:** 2015-02-13

**Authors:** Aubry L. Alvarez, Amy E. Booth

**Affiliations:** ^1^Early Learning Laboratory, Department of Psychology, University of Texas at Austin, Austin, TX, USA; ^2^Department of Communication Sciences and Disorders, University of Texas at Austin, Austin, TX, USA

**Keywords:** causal information, causality, preschoolers, preferences, learning

## Abstract

Young children, in general, appear to have a strong drive to explore the environment in ways that reveal its underlying causal structure. But are they really attuned specifically to casual information in this quest for understanding, or do they show equal interest in other types of non-obvious information about the world? To answer this question, we introduced 20 three-year-old children to two puppets who were anxious to tell the child about a set of novel artifacts and animals. One puppet consistently described causal properties of the items while the other puppet consistently described carefully matched non-causal properties of the same items. After a familiarization period in which children learned which type of information to expect from each informant, children were given the opportunity to choose which they wanted to hear describe each of eight pictured test items. On average, children chose to hear from the informant that provided causal descriptions on 72% of the trials. This preference for causal information has important implications for explaining the role of conceptual information in supporting early learning and may suggest means for maximizing interest and motivation in young children.

## INTRODUCTION

Causal information appears to have special status in the minds of young children. Even infants have a sophisticated sensitivity to the causal structure of their world (e.g., Oakes and Cohen, 1994; Gopnik et al., 2001; Legare et al., 2010; Mascalzoni et al., 2013). Importantly, this sensitivity appears to support early categorization, word learning, and memory more generally speaking (e.g., Bauer and Mandler, 1989; Booth and Waxman, 2002; Booth, 2008, 2009). For example, 3-year-olds are more likely to remember novel labels if their novel animal or artifact referents are described in terms of their causal properties than if they are described in terms of their causally-irrelevant properties (Booth, 2009, 2014; also see Kemler Nelson et al., 2008). But why does causal information have this facilitative effect on learning? One possibility explored in the current work is that causal information taps into children’s natural drive to learn about how and why things behave and interact as they do.

Although the notion of children as little scientists questing for knowledge originated in Piagetian theory (e.g., Piaget, 1952), contemporary researchers have advanced similar ideas. Gopnik (1998, 2000) has gone as far as to suggest that positive physiological sensations propel children to acquire causal knowledge, just as orgasm propels adults to reproduce (see Biederman and Vessel, 2006 for relevant physiological evidence). The intrinsic value conferred upon causal information by such a mechanism, would likely lead children to privilege causal over non-causal inputs. The resulting heightened attention to causal information might further enhance information processing and memory, thereby facilitating learning (e.g., Craik et al., 1996). In the current work, we take a first step toward testing this possibility. We reasoned that if causal information facilitates learning because of its intrinsic interest, then we should be able to observe that interest in children’s preferences.

Ample research already reveals that young children are sensitive to causal information, and that they seek out causal explanations when they are not immediately obvious (e.g., Gopnik and Sobel, 2000; Schulz and Bonawitz, 2007; Asher and Kemler Nelson, 2008; Legare et al., 2010). For example, when given the opportunity to inquire freely about novel objects, preschoolers most often want to know about causally-relevant properties (Kemler Nelson et al., 2004; Greif et al., 2006). Related research also indicates that young children tend to choose explanations involving function (a notably rich causal construct) when asked what they believe objects and their physical parts are “for” (Keil, 1992; Kelemen, 1999). None of this work, however, has explicitly contrasted children’s interests in learning causal vs. non-causal information. In order to provide a direct test of children’s predilection for causal information, we assessed preschoolers’ preferences for hearing from an informant that always provided causal descriptions of novel artifacts and animals versus an informant that always provided equally plausible and distinctive, but non-causal, descriptions of the same items. We reasoned that, if learning causal information is especially valued (e.g., Gopnik, 1998, 2000), children should more often choose to hear the causal descriptions.

## MATERIALS AND METHODS

### PARTICIPANTS

Because we were interested in why causal information might facilitate learning, we focused on 3-year-olds, an age at which this facilitative effect has been robustly observed (e.g., Kemler Nelson et al., 2008; Booth, 2009, 2014). Twenty participants (13 females) were recruited from either a database of Chicago area families or a middle income preschool. Although most parents identified their children as Caucasian, children of African-American (20%), Hispanic (20%), and Asian (5%) descent also participated. Participants (1) fell within an age range of 3;0–3;11 years (*M* = 42.2 mos.; SD = 3.89), (2) demonstrated a receptive vocabulary score within one standard deviation of that expected for their chronological age (Peabody Picture Vocabulary Test-4 (PPVT-4), Form A (Dunn and Dunn, 2007) scores averaged *M* = 101.16, SD = 9.33), (3) had less than 50% daily exposure to a language other than English, and (4) had no parent-reported history of developmental delay or disorder. This research was conducted according to all ethical guidelines provided by the American Psychological Association and with the approval of the institutional review board at Northwestern University.

### MATERIALS

#### Images

**FIGURE 1:**
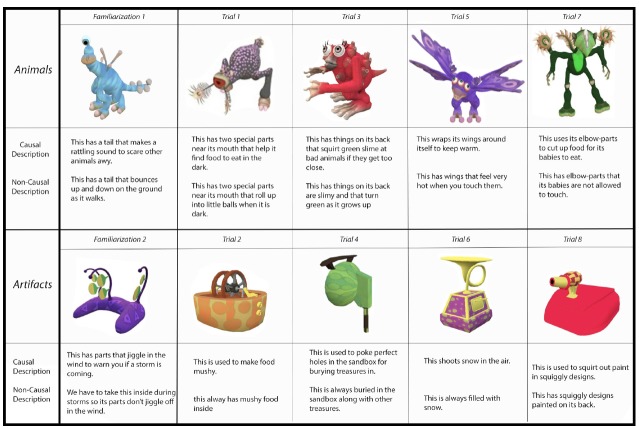
**Visual stimuli with descriptions of their causal and non-causal properties are depicted in the order in which they were presented to children.** Those stimuli depicted in the first column were presented during the familiarization phase. Those depicted in the remaining four columns were presented on trials one through eight of the description-preference phase.

Pictures of ten novel items were generated using Spore^®^ software (see Figure 1). Novel stimuli (rather than existing, real-world items) were used to ensure that familiarity did not influence performance. To provide a broad representation of novel items, and following on prior research examining the facilitative effect of causal information on learning (e.g., Booth, 2009), half of the stimuli were animals and half were artifacts. Each picture was approximately 2 × 3-inches in dimension and was presented in isolation on its own 3 × 5-inch flashcard.

#### Descriptions

Two brief descriptions of non-obvious properties of each pictured item were developed similar to those used in Booth (2009). One description highlighted a causally-relevant property that reflected how the object (or a part thereof) is used to achieve the goals of a human or non-human agent (See Figure 1). The other description also highlighted a non-obvious property, but did not provide any hints regarding function. Although it could be argued that these latter descriptions were not truly devoid of causal information in that some might invite relevant inferences, they were relatively impoverished in this respect, focusing instead on perceptual or other properties that did not provide any conceptual insight. Note that to the extent that these descriptions did embody causal information, or support inferences thereof, they would weaken the strength of our manipulation and would therefore work against our hypothesis.

Causal and non-causal descriptions were matched as closely as possible in terms of length, composition, plausibility and distinctiveness. Specifically, descriptions were matched on total number of words (*M*_causal_ = 12.6 vs. *M*_non-causal_ = 12.1) and syllables (*M*_causal_ = 14.9 vs. *M*_non-causal_ = 14.6), as well as the number of nouns, adjectives, and verbs they included. Furthermore, 12 undergraduates were asked to (1) rate each description in terms of its plausibility with respect to its associated novel item and (2) list all real-world items that they could think of that fit each description. Conditions were equivalent in terms of both plausibility (*M*_causal_ = 3.6 vs. *M*_non-causal_ = 3.6) and distinctiveness (*M*_causal_ = 1.41 vs. *M*_non-causal_ = 1.34).

In some respects it would have been ideal to match the descriptions even further in terms of the specific content words included therein. For example, the causal description, “This has two special parts near its mouth that help it find food to eat in the dark” might be matched with the non-causal description, “This can find things to eat in the dark *and* it has two special things near its mouth.” Although, whenever possible, we used the same content words across causal and non-causal descriptions, a fuller implementation of this type of precise matching was problematic for two reasons. First, the *and* conjunction is generally less constraining on the distinctiveness of the descriptions than is a causal bridge, thus forcing a mismatch on another important control dimension. Second, we felt that linking the two elements of each descriptor with the *and* conjunction might well facilitate children’s spontaneous inference of a causal link between those elements. Because minimizing the causal composition of the non-causal descriptions was critical to our design, we felt that this strategy was therefore untenable for the current investigation. We do not believe that this unduly weakens the strength of our manipulation because there is no obvious systematic reason why the mismatched content words used in one condition would be more attractive than those used in the other. Moreover, and of critical importance, children made their selections on each trial *prior* to hearing each pair of descriptions. Thus, selections were necessarily based on which speaker reliably provided which type of information, rather than on a direct comparison of the descriptions provided on each trial.

#### Puppet informants

Two dog puppets, wearing different colored accessories, served as informants. Nearly identical puppets were used to minimize the likelihood that children’s choices would be influenced by their opinion of the individual informants. Which informant was assigned to provide the causal descriptions was counterbalanced across subjects.

### PROCEDURE

The experiment was conducted during a single session either at the Early Learning Laboratory at Northwestern University or at the participant’s Chicago area preschool. Participants sat opposite the experimenter at a small table. Sessions were recorded via a Panasonic SDR-S26 camera placed approximately six feet across from the participant. Each participant completed a familiarization phase, a description-preference test phase and an informant-preference post-test phase.

#### Familiarization phase

The experimenter began by telling the child that they were going to play a game in which two puppets would tell them different kinds of things about a picture. The experimenter asked the participant to listen closely. For each of two familiarization trials, the experimenter first placed a picture of a novel artifact or animal on the table. She then provided both the causal and non-causal descriptions thereof, one via each puppet informant. Which description type was provided first was counterbalanced across familiarization trials and participants. Additionally, the location of the causal and non-causal informants was counterbalanced across participants. Children had approximately 15 s to visually inspect each picture. At the end of the familiarization phase, the experimenter reminded the participant that each puppet provided different types of information, and told them that they would now be allowed to choose which one they wanted to tell them about each picture.

#### Description-preference test phase

For each of the eight trials, the experimenter showed the participant a picture of a novel item and provided either the causal or non-causal description according to the child’s choice of informant. In order to minimize the likelihood that children would make choices based on novelty relative to the preceding trial(s), the experimenter then provided the non-preferred description of the item via the unselected informant.

#### Informant-preference post-test phase

As a check on the possibility that children’s choices might be determined by preferences for a particular informant (irrespective of the type of information they provided), the experimenter asked the participants which puppet they preferred at the conclusion of the experiment. Importantly, no new picture was provided at this time, making it clear to children that they should not expect any new information based on their selection.

## RESULTS

Our primary dependent variable of interest was the proportion of trials (out of eight) on which participants chose to hear from the informant that always provided causal descriptions. Participants chose the causal informant on an average of 72% of trials (SD = 0.21), a level of preference that differed significantly from chance, *t*(19) = 15.33, *p* < 0.001, *d* = 7.03. Individual difference analyses mirrored this finding, with 15 children preferring to hear from the causal informant first on the majority of trials in contrast to only two preferring to hear from the non-causal informant first on the majority of trials, Yates’ χ^2^ (1, *N* = 17) = 4.17, *p* = 0.04. The remaining three children chose the causal and non-causal informants an equal number of times. On a trial by trial basis, the proportion of children selecting the causal informant ranged from 0.5 to 0.9, with no significant changes observed across any two consecutive trials, except from trial 5 (0.85) to trial 6 (0.5), McNemar’s χ^2^ (1, *N* = 20) = 4.00, *p* = 0.05.

Importantly, test trial choices did not appear to stem from a preference for one informant over the other (irrespective of the type of descriptions they provided). When simply asked which informant they liked best (in the absence of a novel picture and imminent description thereof), just over half of the children (*n* = 12) preferred the causal informant, and three of these were children who had actually shown a preference for the non-causal informant during the preceding test trials. The remaining children preferred the non-causal informant (*n* = 6) or indicated no preference (*n* = 2) in the post-test. Further reflecting the lack of correspondence between preferences for description-type and informant is the fact that children requested causal information with equal frequency during description-type testing regardless of which informant they chose during the post-test (*M* = 5.75, SD = 2.0 vs. *M* = 5.63, SD = 1.33).

Although not central to our predictions, we also examined the data for potential effects of stimulus domain. A paired *t*-test comparing preferences on artifact versus animate kind trials revealed no domain differences (*M*_animate_ = 0.76, SD = 0.27; *M*_artifact_ = 0.71, SD = 0.25), *t*(19) = 0.64, *p* = 0.53.

## DISCUSSION

In sum, when given the choice between hearing causal versus non-causal descriptions of novel artifacts and animals, preschoolers preferred the former. Importantly, this preference was not driven by which informant children liked best. These results demonstrate that children distinguish between different types of descriptive information about unfamiliar objects and animals, and show a clear preference for causally-relevant information from an early age. This conclusion is consistent with the theoretical position that children are highly motivated to learn about the causal structure of their world (Piaget, 1952; Gopnik, 1998). The current findings are also consistent with empirical work indicating that infants and young children actively seek out and use information regarding novel causal properties (e.g., Gopnik and Sobel, 2000; Booth, 2009; Schulz and Bonawitz, 2007; Legare et al., 2010). Although children’s particular interest in causality is strongly implied by this literature, the current study is the first to provide a direct test of children’s preferences by pitting causal information against carefully matched alternatives.

One might argue that the current results could be alternatively interpreted in terms of children’s well-established preferences for knowledgeable speakers (e.g., Koenig et al., 2004; Corriveau and Harris, 2009). That is, although the procedure offered no explicit reason for children to suspect the informants to have different levels of knowledge, children might have perceived the causal informant as more knowledgeable based solely on the type of information it provided. Indeed, Sobel and Corriveau (2010) report that 4-year-olds are more likely to endorse labels for novel objects from speakers who previously demonstrated more causal knowledge about those objects. Importantly, however, 4-year-old participants (equivalent in age to those tested here) did not respond systematically in Sobel and Corriveau’s task. Thus, it seems more likely that participants in the current study based their selections on the type of information that they expected to receive rather than on the perceived reliability or trustworthiness of the individual informants.

One might also question whether some unintentional difference between the causal and non-causal descriptions might account for children’s preferences. For example, while well matched in terms of length and content, descriptions were not explicitly matched across conditions in terms of the degree to which they detailed the dynamic activity of their referent. Although this dimension is difficult to quantify, it is clear that while all of the causal descriptions included a dynamic component, only some of the non-causal descriptions did. Indeed, two non-causal descriptions were particularly devoid of dynamic activity (“This always has mushy food inside” and “This is always filled with snow”). Children did not, however, select the causal informant at unusually high rates on the trials immediately following presentation of these static non-causal descriptions. The proportion of children selecting the causal informant was 0.70 for both of these trials, a value that fell precisely at the median across all trials. That said, further investigation will be required to definitively determine whether the dynamic quality of causal descriptions contributes to their being preferred by young children.

Importantly, not only are the current results revealing about children’s preferences, but they also offer potential insight into why causal information facilitates learning in young children. For example, they suggest that 3-year-olds might better remember novel words when provided with causal rather than non-causal descriptions of their referents because the causal descriptions are particularly interesting. In this way, causal information might harness children’s attention during the learning task, thereby facilitating information processing. The current investigation of course did not directly measure the effects of causal information on attention, leaving underspecified an important link in the logic undergirding this proposed mechanism (Booth, 2009, 2014). Given that prior studies have not reported differences in outward signs of attention in experimental conditions that include causal information (e.g., Booth, 2009), it is likely that more sensitive measures like heart-rate will be required to illustrate (or refute) this link.

Additional inquiry will also be required to further specify the generalizability of the results reported here. For example, it remains to be specified when causal information comes to be preferred by children. Is it a biologically determined predisposition, or is it something that is shaped by experience, only emerging in the preschool years? Recent research suggests that preferences for physically causal events are present even in newborn infants (Mascalzoni et al., 2013). However, that work focuses on a very different form of causal information than that investigated here. Indeed there are numerous ways in which causal information might be instantiated, and moving forward, it will be crucial to consider these multiple forms developmentally, as well as across different contexts (e.g., physical object exploration, book reading, group play in preschools).

In closing, it is worth noting that the current findings also have potentially important practical implications. Specifically, highlighting the causal relevance of information might enhance learning in early childhood education settings. Evidence suggests that causal information itself is not only learned more effectively by children than non-causal information, but that other perceptual and linguistic information presented along with causal information may also benefit by virtue of the advantageous mental state it induces (Subramaniam et al., 2009; Booth, 2014). Thus, integrating causal information into otherwise less engaging learning tasks has the potential to have a significant impact. Forging bridges between basic research like that reported here, and educational practice will therefore be an important priority as we advance our understanding of the role that causal information plays in supporting cognitive development.

### Conflict of Interest Statement

The authors declare that the research was conducted in the absence of any commercial or financial relationships that could be construed as a potential conflict of interest.

## References

[B1] Asher Y. M. Kemler Nelson D. G. (2008). Was it designed to do that? Children’s focus on intended function in their conceptualization of artifacts. Cognition 106, 474–483. 10.1016/j.cognition.2007.01.007 17331491

[B2] Bauer P. J. Mandler J. M. (1989). One thing follows another: effects of temporal structure on 1- and 2-year-olds’ recall of events. Dev. Psychol. 25, 197–206 10.1037/0012-1649.25.2.197

[B3] Biederman I. Vessel E. (2006). Perceptual pleasure and the brain a novel theory explains why the brain craves information and seeks it through the senses. Am. Sci. 94, 247 10.1511/2006.3.247

[B4] Booth A. E. (2008). The cause of infant categorization? Cognition 106, 984–993. 10.1016/j.cognition.2007.03.012 17459362

[B5] Booth A. E. (2009). Causal supports for early word learning. Child Dev. 80, 1243–1250. 10.1111/j.1467-8624.2009.01328.x 19630905 PMC2786819

[B6] Booth A. E. (2014). Effects of causal information on early word learning: efficiency and longevity. Cogn. Dev. 33, 99–107 10.1016/j.cogdev.2014.05.001

[B7] Booth A. E. Waxman S. (2002). Object names and object functions serve as cues to categories for infants. Dev. Psychol. 38, 948–957. 10.1037/0012-1649.38.6.948 12428706

[B8] Corriveau K. Harris P. L. (2009). Choosing your informant: weighing familiarity and recent accuracy. Dev. Sci. 12, 426–437. 10.1111/j.1467-7687.2008.00792.x 19371367

[B9] Craik F. I. M. Govoni R. Naveh-Benjamin M. Anderson N. D. (1996). The effects of divided attention on encoding and retrieval processes in human memory. J. Exp. Psychol. 125, 159–180 10.1037/0096-3445.125.2.159 8683192

[B10] Dunn L. M. Dunn D. M. (2007). Peabody Picture Vocabulary Test, Fourth Edition. San Antonio, TX: Pearson.

[B11] Gopnik A. (1998). Explanation as orgasm. Minds Mach. 8, 101–118 10.1023/A:1008290415597

[B12] Gopnik A. (2000). “Explanation as orgasm and the drive for causal knowledge: the function, evolution, and phenomenology of the theory formation system,” in Explanation and Cognition, eds Keil F. C. Wilson R. A. (Cambridge, MA: MIT Press), 299–323.

[B13] Gopnik A. Sobel D. (2000). Detecting blickets: how young children use information about novel causal powers in categorization and induction. Child Dev 71, 1205–1222. 10.1111/1467-8624.00224 11108092

[B14] Gopnik A. Sobel D. Schulz L. E. Glymour C. (2001). Causal learning mechanisms in very young children: two-, three-, and four- year olds infer causal relations from patterns of variation and covariation. Dev. Psychol. 37, 620–629. 10.1037/0012-1649.37.5.620 11552758

[B15] Greif M. L. Nelson D. G. K. Keil F. C. Gutierrez F. (2006). What do children want to know about animals and artifacts? Domain-specific requests for information. Psychol. Sci. 17, 455–459. 10.1111/j.1467-9280.2006.01727.x 16771792 PMC3034738

[B16] Keil F. C. (1992). “The origins of an autonomous biology,” in Modularity and Constraints in Language and Cognition, Minnesota Symposia on Child Psychology. Vol. 25, eds Garman M. R. Maratsos M. (Hillsdale, NJ: Erlbaum), 103–138.

[B17] Kelemen D. (1999). The scope of teleological thinking in preschool children. Cognition 70, 214–272 10.1016/S0010-0277(99)00010-4 10384737

[B18] Kemler Nelson D. G. Chan E. L. Holt M. B. (2004). When children ask, “What is it?” what do they want to know about artifacts?. Psychol. Sci. 15, 384–389. 10.1111/j.0956-7976.2004.00689.x 15147491

[B19] Kemler Nelson D. G. O’Neill K. Asher Y. M. (2008). A mutually facilitative relationship between learning names and learning concepts in preschool children: the case of artifacts. J. Cogn. Dev. 9, 171–193 10.1080/15248370802022621

[B20] Koenig M. A. Clement F. Harris P. (2004). Trust in testimony: children’s use of true and false statements. Psychol. Sci. 15, 694–698. 10.1111/j.0956-7976.2004.00742.x 15447641

[B21] Legare C. H. Gelman S. A. Wellman H. M. (2010). Inconsistency with prior knowledge triggers children’s causal explanatory reasoning. Child Dev. 81, 929–944. 10.1111/j.1467-8624.2010.01443.x 20573114 PMC3039682

[B22] Mascalzoni E. Regolin L. Vallortigara G. Simion F. (2013). The cradle of causal reasoning: newborns’ preference for physical causality. Dev. Sci. 16, 327–335. 10.1111/desc.12018 23587033

[B23] Oakes L. M. Cohen L. B. (1994). “Infant causal perception,” in Advances in Infancy Research, Vol. 9, eds Rovee-Collier C. Lipsitt L. P. (Norwood, NJ: Ablex Publishing Corporation), 1–54.

[B24] Piaget J. (1952). The Origins of Intelligence in Children. New York, NY: International Universities Press.

[B25] Schulz L. E. Bonawitz E. B. (2007). Serious fun: preschoolers engage in more exploratory play when evidence is confounded. Dev. Psychol. 43, 1045–1050. 10.1037/0012-1649.43.4.1045 17605535

[B26] Sobel D. M. Corriveau K. H. (2010). Children monitor individuals’ expertise for word learning. Child Dev. 81, 669–679. 10.1111/j.1467-8624.2009.01422.x 20438467

[B27] Subramaniam K. Kounios J. Bowden E. M. Parrish T. B. Jung-Beeman M. (2009). A brain mechanism for facilitation of insight by positive affect. J. Cogn. Neurosci. 21, 415–432. 10.1162/jocn.2009.21057 18578603

